# Differential renal effects of candesartan at high and ultra-high doses in diabetic mice–potential role of the ACE2/AT2R/Mas axis

**DOI:** 10.1042/BSR20160344

**Published:** 2016-10-27

**Authors:** Glaucia E. Callera, Tayze T. Antunes, Jose W. Correa, Danielle Moorman, Alexey Gutsol, Ying He, Aurelie Nguyen Dinh Cat, Ana M. Briones, Augusto C. Montezano, Kevin D. Burns, Rhian M. Touyz

**Affiliations:** * Kidney Research Centre, Ottawa Hospital Research Institute, University of Ottawa, Ottawa, Ontario, Canada K1H 8M5; † Institute of Cardiovascular & Medical Sciences, BHF Glasgow Cardiovascular Research Centre, University of Glasgow, Glasgow, Canada G12 8TA

**Keywords:** albuminuria, candesartan, nephropathy, RAS, type 2 diabetes

## Abstract

High doses of Ang II receptor (AT1R) blockers (ARBs) are renoprotective in diabetes. Underlying mechanisms remain unclear. We evaluated whether high/ultra-high doses of candesartan (ARB) up-regulate angiotensin-converting enzyme 2 (ACE2)/Ang II type 2 receptor (AT2R)/Mas receptor [protective axis of the of the renin–angiotensin system (RAS)] in diabetic mice. Systolic blood pressure (SBP), albuminuria and expression/activity of RAS components were assessed in diabetic db/db and control db/+ mice treated with increasing candesartan doses (intermediate, 1 mg/kg/d; high, 5 mg/kg/d; ultra-high, 25 and 75 mg/kg/d; 4 weeks). Lower doses candesartan did not influence SBP, but ultra-high doses reduced SBP in both groups. Plasma glucose and albuminuria were increased in db/db compared with db/+ mice. In diabetic mice treated with intermediate dose candesartan, renal tubular damage and albuminuria were ameliorated and expression of ACE2, AT2R and Mas and activity of ACE2 were increased, effects associated with reduced ERK1/2 phosphorylation, decreased fibrosis and renal protection. Ultra-high doses did not influence the ACE2/AT2R/Mas axis and promoted renal injury with increased renal ERK1/2 activation and exaggerated fibronectin expression in db/db mice. Our study demonstrates dose-related effects of candesartan in diabetic nephropathy: intermediate–high dose candesartan is renoprotective, whereas ultra-high dose candesartan induces renal damage. Molecular processes associated with these effects involve differential modulation of the ACE2/AT2R/Mas axis: intermediate–high dose candesartan up-regulating RAS protective components and attenuating pro-fibrotic processes, and ultra-high doses having opposite effects. These findings suggest novel mechanisms through the protective RAS axis, whereby candesartan may ameliorate diabetic nephropathy. Our findings also highlight potential injurious renal effects of ultra-high dose candesartan in diabetes.

## INTRODUCTION

Major morbidities associated with diabetes include adverse cardiovascular events and nephropathy, a leading cause of end-stage renal disease [1]. The renin–angiotensin system (RAS) plays a pivotal role in the pathophysiology of cardiovascular and kidney dysfunction. This is supported by extensive experimental and clinical data demonstrating that RAS inhibition, besides reducing blood pressure, slows progression of proteinuric kidney disease and protects against target organ damage [2–14]. The RAS, in its classic definition, is known as an endocrine system that exerts its actions through the effector peptide angiotensin II (Ang II). Besides being a potent vasoactive peptide and regulator of salt and volume homoeostasis, Ang II, through its type 1 receptor (AT1R) promotes target organ damage by activating proinflammatory, pro-fibrotic and mitogenic signalling pathways [12–14]. Other components of the RAS may counteract these Ang II/AT1R damaging actions, such as signalling through the Ang II type 2 receptor (AT2R). In addition Ang-(1–7), produced by angiotensin-converting enzyme 2 (ACE2), and which signals through the Mas receptor, has been associated with cardiovascular and renal protection, in part, by antagonizing Ang II/AT1R actions [15–19].

Growing evidence indicates that ACE inhibitors and AT1R blockers (ARBs) protect against diabetic kidney injury independent of their haemodynamic effects [2,3]. Clinical studies have shown beneficial effects of ACE inhibitors and ARBs in delaying the onset of microalbuminuria in patients with type 2 diabetes [20–22]. Combined therapy with ACE inhibitors and ARBs decreases proteinuria and further slows the progression of glomerular filtration rate decline [22–26]. However, these observations have been challenged in light of new findings from large clinical studies [27,28]. Patients exhibited worsening of renal outcomes with combined ACE inhibitors and ARBs, including an increased risk of hyperkalaemia and acute kidney injury [27,28]. On the other hand, significant renal protection by ARB monotherapy was observed when patients with diabetes were treated with higher doses than those recommended as antihypertensive treatment [29–33]. The beneficial effects of high dose ARBs have been attributed, in part, to reduction in glomerular capillary hydrostatic pressure, decreased expression of proinflammatory mediators and reduced oxidative stress [33,34]. However molecular mechanisms underlying these phenomena are unclear and it is unknown whether high-dose ARBs influence the ‘protective’ components of the RAS, namely AT2R, ACE2 and Ang-(1–7)/Mas.

Since proteinuria predicts adverse renal outcomes and conversely its reduction is associated with improved kidney function [35], we evaluated whether the AT1R antagonist candesartan, in an intermediate- to ultra-high dose range, reduces renal injury in db/db mice, a model of type 2 diabetes. We also tested whether increasing doses of candesartan up-regulates renal AT2R, ACE2 and Ang-(1–7)/Mas expression/activity.

## MATERIALS AND METHODS

See supplementary text for extended methods section.

### Animals

Seven-week-old male Lepr^db^/Lepr^+^ (db/+) and Lepr^db^/Lepr^db^ (db/db) mice (from Jackson Laboratories) were treated for 4 weeks (by subcutaneous injection) with increasing doses of candesartan (AstraZeneca): intermediate (1 mg/kg/d), high (5 mg/kg/d) and ultra-high (25 and 75 mg/kg/d). Vehicle-treated db/db and db/+ mice were used as control groups. Systolic blood pressure (SBP) was measured by tail-cuff plethysmography.

### Urinary albumin and creatinine excretion

Spot urine was collected for assessment of albumin (μg/ml) and creatinine (mg/ml). Results were expressed as albumin:creatinine ratio (ACR, μg/mg).

### Western blot

Total protein was extracted from kidney cortex and probed for AT2R, Mas, ACE2, ERK1/2, p38MAPK, JNK, plasminogen activator inhibitor-1 (PAI-1), vascular cell adhesion molecule-1 (VCAM-1) and osteopontin (OPN) by western blotting.

### Kidney staining and morphometry

Fixed paraffin-embedded kidney tissues were stained with haematoxylin & eosin and periodic acid–Schiff (PAS) staining.

### ACE and ACE2 enzymatic activity

ACE and ACE2 enzymatic activity in plasma and kidney cortex homogenates were determined following incubations with the respective synthetic specific substrates.

### AT1R expression by quantitative real-time PCR

Total RNA was extracted from kidney cortex. Real-time PCR was used to determine the expression levels of AT1R mRNA.

### Statistical analysis

Data are presented as means ± S.E.M. Groups were compared using one-way ANOVA with Tukey correction to compensate for multiple testing procedures and two-way ANOVA with Bonferroni post-hoc test, as appropriate. *P*<0.05 was significant.

## RESULTS

### Animal data and plasma analyses

Body mass and relative kidney mass were greater in db/db mice compared with db/+ mice and were unaffected by candesartan treatment (Supplementary Table S1). Plasma biochemistry is summarized in Table 1. In vehicle-treated groups plasma glucose, phosphate and creatinine levels were higher in db/db mice compared with db/+ mice, without any effect of treatment. Candesartan at 25 and 75 mg/kg/d increased plasma phosphate and creatinine in db/+ mice compared with vehicle-treated db/+ counterparts. In vehicle-treated groups blood urea nitrogen (BUN) levels were similar in db/db mice and db/+ mice. BUN progressively increased in both experimental groups with candesartan treatment.

**Table 1:** Plasma metabolic panel in db/+ and db/db mice treated with candesartan *P<0.05 compared with vehicle-treated db/+; **P<0.05 db/db compared with db/+ of corresponding candesartan dose. *n*=5–7/group.

	Vehicle		1 mg/kg/d		5 mg/kg/d		25 mg/kg/d		75 mg/kg/d	
	db/+	db/db	db/+	db/db	db/+	db/db	db/+	db/db	db/+	db/db
Phosphorus (mmol/l)	1.67±0.11	2.50±0.10*	1.77±0.12	2.46±0.19*^,^**	1.83±0.05	2.69±0.31*^,^**	2.19±0.03*	2.58±0.18*	2.04±0.15*	2.88±0.54*
Creatinine (μmol/l)	24.7±1.6	35.7±3.6*	25.6±1.2	35.7±2.3*	27.0±2.0	33.2±2.2*	30.2±2.3*	35.0±2.1*	35.7±4.1*	41.2±0.9*
BUN (mmol/l)	6.9±0.2	8.7±1.8	7.5±0.4	10.2±1.0	11.7±0.1*	9.1±0.5	30.0±4.1*	22.5±9.0	46.1±12.4*	23.1±7.1
BUN/creatinine (mg/dl)	71.29±5.2	60.33±8.6	73.76±7.0	72.13±6.6	111.1±14.2*	70.06±7.4**	246.2±22.6*	162.0±65.7*	302.7±51.3*	137.2±40.2**
Cholesterol (mmol/l)	1.88±0.06	2.31±0.28	2.04±0.10	2.09±0.21	2.26±0.07	2.06±0.19	2.46±0.12	2.0±0.16	2.38±0.17	2.59±0.18
Glucose (mmol/l)	13.6±0.9	46.4±4.8*	13.6±1.3	46.4±6.3*^,^**	12.5±0.7	51.4±5.3*^,^**	14.2±0.6	67.6±10.4*^,^**	12.2±0.7	60.3±8.4 *^,^**

### Systolic blood pressure

**Figure 1:**
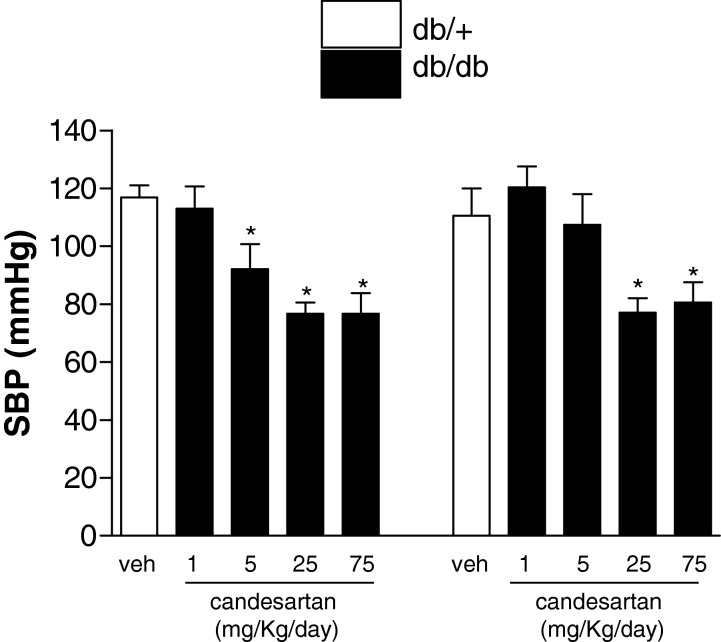
Ultra-high dose of candesartan reduce SBP in db/+ and db/db mice End point SBP in db/+ and db/db mice treated with subcutaneous injections of vehicle or candesartan at 1 mg/kg/d, 5 mg/kg/d, 25 mg/kg/d, 75 mg/kg/d for 4 weeks. Results are mean ± S.E.M. of seven mice. *P<0.05, candesartan-treated compared with counterpart vehicle-treated mice.

Figure 1 shows SBP in mice at the study end point. At 4 weeks of treatment, 1 mg/kg/d candesartan had no effect on SBP in db/+ or db/db mice. A reduction in SBP was observed with 5 mg/kg/d candesartan after 4 weeks treatment in the db/+ mice compared with vehicle-treated db/+ mice. The hypotensive response was similar in the db/+ and db/db mice at 25 and 75 mg/kg/d candesartan. Time course of decrease in SBP is shown in Supplementary Figure S1. Candesartan evoked a gradual decrease in SBP starting earlier and with lower doses in db/+ mice. Candesartan at 75 mg/kg/d induced a similar time course of decrease in SBP in both experimental groups. No differences were observed in SBP between vehicle-treated db/+ and db/db groups.

### Urinary albumin excretion

**Figure 2:**
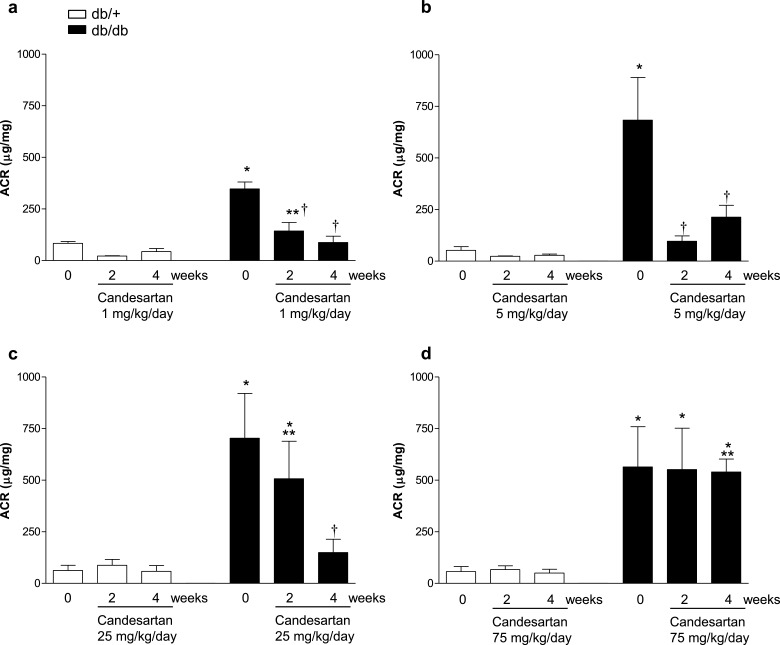
Intermediate to high, but not ultra-high, dose of candesartan ameliorates albuminuria in db/db mice Urinary albumin was assessed in db/+ and db/db mice at baseline (0 time point) and following 2 and 4 weeks of vehicle or candesartan daily subcutaneous injections. Mice were treated with vehicle or candesartan at 1 mg/kg/d (a); 5 mg/kg/d (b); 25 mg/kg/d (c); 75 mg/kg/d (d). Albuminuria was expressed as albumin (μg/ml):creatinine (mg/ml) ratio. Results are presented as mean ± S.E.M. of 5–7 mice in each group. *P<0.05 compared with vehicle-treated db/+; **P<0.05 candesartan-treated db/db compared with db/+ of corresponding week; ^†^*P*<0.05 candesartan-treated db/db compared with vehicle-treated db/db.

The urinary ACR, an index of kidney injury, was increased in db/db mice at baseline compared with db/+ mice (Figure 2). In the vehicle-treated db/db group, the ACR remained elevated for the duration of the study (Supplementary Figure S2). Candesartan at 1 mg/kg/d (Figure 2a), 5 mg/kg/d (Figure 2b) and 25 mg/kg/d (Figure 2c), reduced albuminuria. Higher doses of candesartan had no effect on ACR (Figure 2d).

### Effects of candesartan on kidney histology

**Figure 3:**
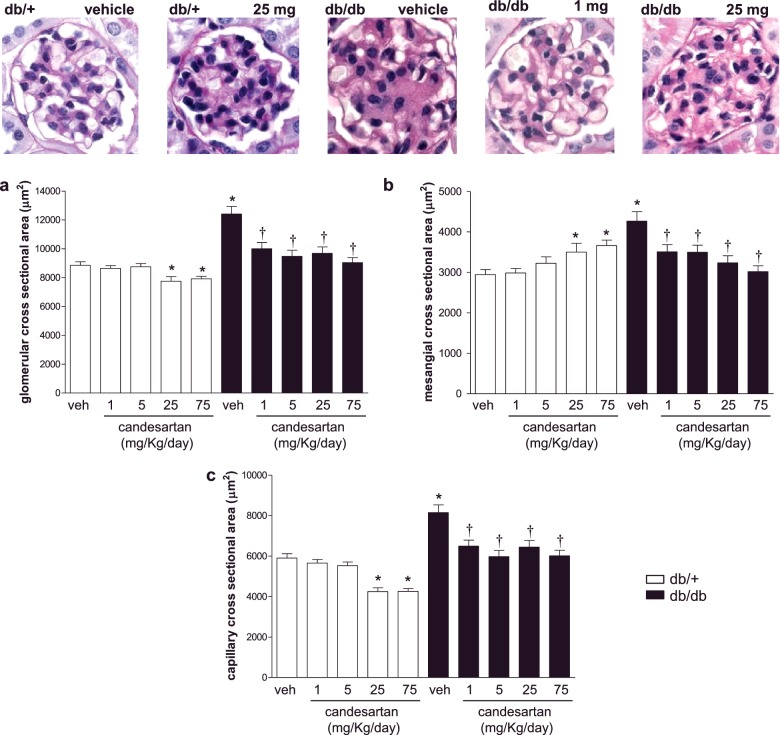
Increasing doses of candesartan reduce glomerular area enlargement in the kidney cortex of db/db mice Kidney morphometry. Glomerular (**a**), mesangial (**b**) and capillary (**c**) cross-sectional area were evaluated in kidney cortex from db/+ and db/db mice treated with vehicle or candesartan 1 mg/kg/d, 5 mg/kg/d, 25 mg/kg/d, 75 mg/kg/d. *P<0.05 compared with vehicle-treated db/+; ^†^*P*<0.05 candesartan-treated db/db compared with vehicle-treated db/db.

**Figure 4:**
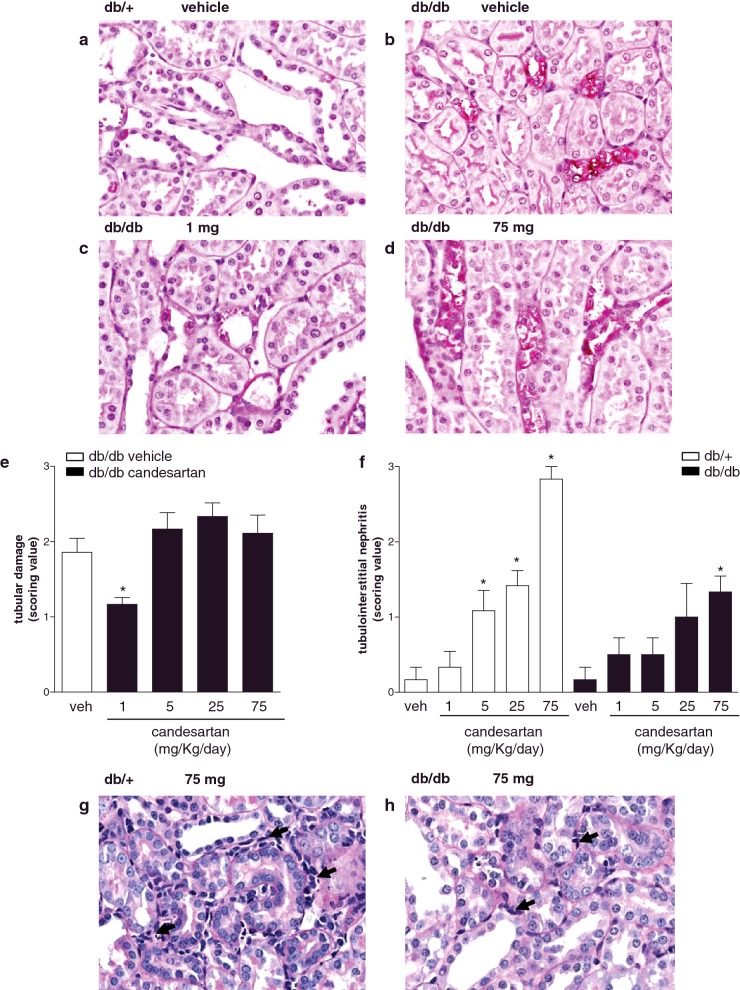
Candesartan (1 mg/kg/d) improves tubular damage in db/db mice Mice were daily treated with subcutaneous injections of vehicle or candesartan (1, 5, 25 or 75 mg/kg/d) for 4 weeks. Representative photographs of PAS-stained tubules and interstitium (**a**–**d**, **g** and **h**). Bar graphs: scores for tubular damage in db/db mice (**e**) and tubulointerstitial nephritis in db/+ and db/db mice (**f**). Arrows, indicate cell infiltrates. *P<0.05 compared with vehicle-treated counterpart.

Histological features of kidney structure in db/db and db/+ mice are presented in Figure 3. db/db mice showed increased glomerular, mesangial and capillary cross-sectional area, compared with db/+ mice, effects that were reduced by candesartan (Figures 3a–3c). In db/+ mice, candesartan ultra-high doses significantly reduced glomerular and capillary cross-sectional area and increased mesangial cross-sectional area. db/db and db/+ mice did not exhibit differences in mesangial cell number per glomerular section (Supplementary Figure S3). Features of nephropathy are shown in Figure 4. In vehicle-treated db/db mice, distal tubules showed dystrophic lesions with areas of complete desquamation and necrosis (Figure 4b). Only 1 mg/kg/d candesartan was effective in decreasing distal tubular damage in db/db mice (Figures 4c–4e). Candesartan induced cortical tubulointerstitial nephritis in a dose-dependent manner in both groups (Figure 4f). Focal tubular basophilia, at S1–S2 segments of the proximal tubules of the outer cortex, was predominant in the subcapsular zone (Figures 4g and 4h). Cell infiltrates comprised rare neutrophils and lymphocytes with predominant macrophages. There was no evidence of ischemic tubular injury as there were no signs of epithelial necrosis of the straight segments of the proximal tubules and the ascending limbs of the loops of Henle.

### Effects of candesartan on RAS receptor expression in kidney cortex

**Figure 5:**
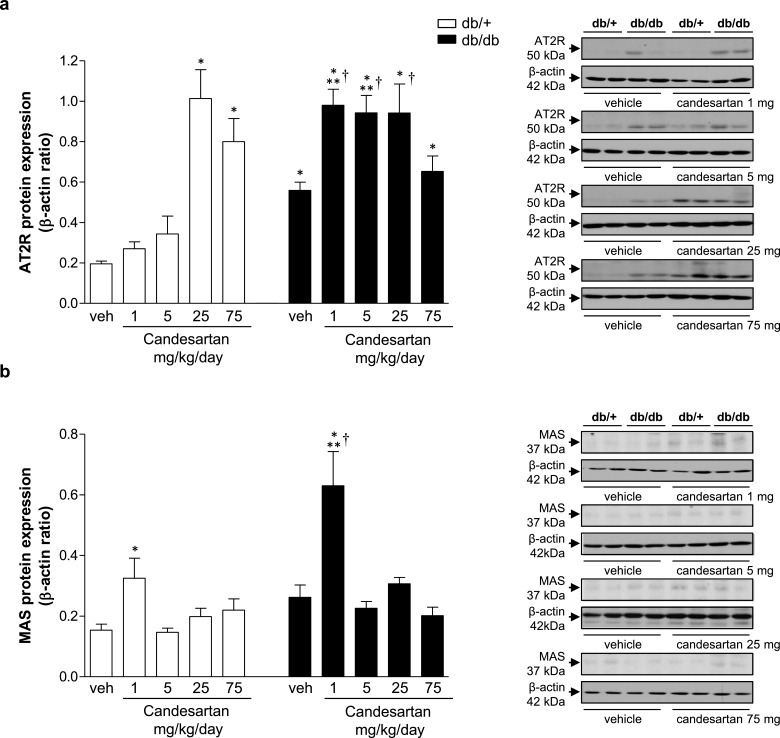
Candesartan modulates expression of RAS receptors in kidney cortex from db/db mice Protein expression of AT2R (**a**) and Mas receptor (**b**) in kidney cortex from db/+ and db/db mice treated with vehicle or candesartan (1, 5, 25 or 75 mg/kg/d). Side panels, representative immunoblots of AT2R, Mas receptor and β-actin. Results are presented as mean ± S.E.M. of 5–7 mice. *P<0.05 compared with vehicle-treated db/+; **P<0.05 db/db compared with db/+ of corresponding candesartan dose; ^†^*P*<0.05 candesartan-treated db/db compared with vehicle-treated db/db.

In vehicle-treated groups, db/db mice displayed increased expression of AT2R compared with db/+ mice (Figure 5a). In db/db mice, candesartan (1–25 mg/kg/d) produced an additional increase in AT2R expression. In db/+ mice, an increase in AT2R expression was observed only at ultra-high candesartan doses. Mas receptor expression was similar in vehicle-treated db/+ and db/db mice (Figure 5b). Treatment with 1 mg/kg/d candesartan increased expression of Mas receptor in both groups.

AT1R expression was studied at the transcript level, due to the fact that commercial AT1R antibodies are non-specific [36]. Kidney AT1R mRNA expression was similar in db/+ and db/db mice treated with vehicle (Supplementary Figure S4). In db/db mice, candesartan at 1 and 75 mg/kg/d decreased mRNA expression of AT1R, whereas no effects were observed at other doses. Candesartan did not affect AT1R mRNA expression in db/+ mice.

### Effects of candesartan on expression and activity of ACE and ACE2

**Figure 6:**
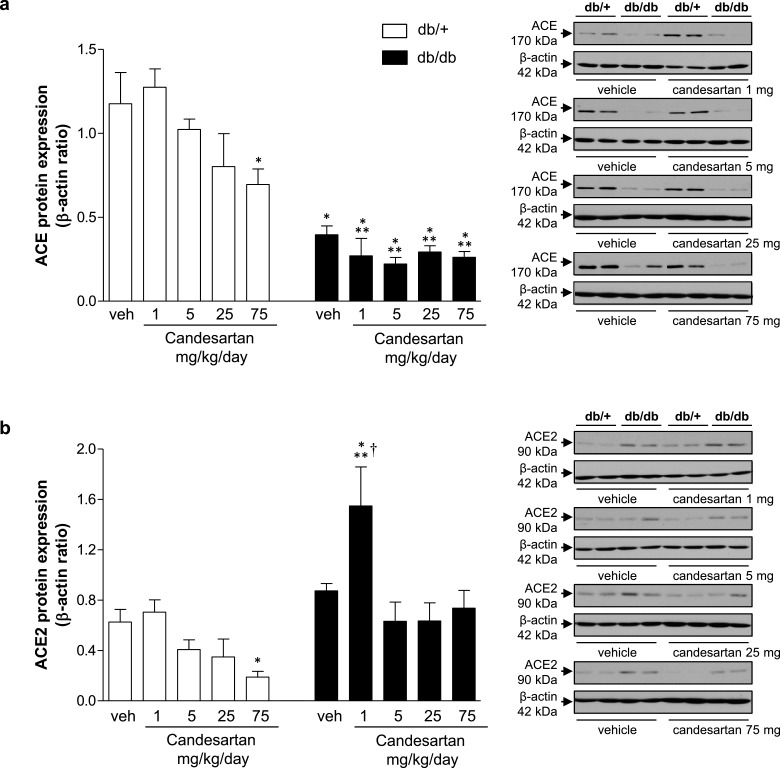
Intermediate dose of candesartan increases expression of ACE2 in kidney cortex from db/db mice Protein expression of ACE (**a**) and ACE2 (**b**) in kidney cortex from db/+ and db/db mice treated with vehicle or candesartan (1, 5, 25 or 75 mg/kg/d). Side panels, representative immunoblots of ACE, ACE 2 and β-actin. Results are presented as mean ± S.E.M. of 5–7 mice. *P<0.05 compared with vehicle-treated db/+; **P<0.05 db/db compared with db/+ of corresponding candesartan dose; ^†^*P*<0.05 candesartan-treated db/db compared with vehicle-treated db/db.

As shown in Figure 6(a), ACE expression in kidney cortex was lower in all db/db groups compared with vehicle- and candesartan-treated counterparts. Figure 6(b) shows similar ACE2 expression in kidney from vehicle-treated db/+ and db/db mice. At 1 mg/kg/d, candesartan increased ACE2 expression in db/db mice compared with vehicle-treated db/db mice. Candesartan at ultra-high dose, reduced expression of ACE and ACE2 in db/+ mice compared with vehicle-treated counterparts.

**Figure 7:**
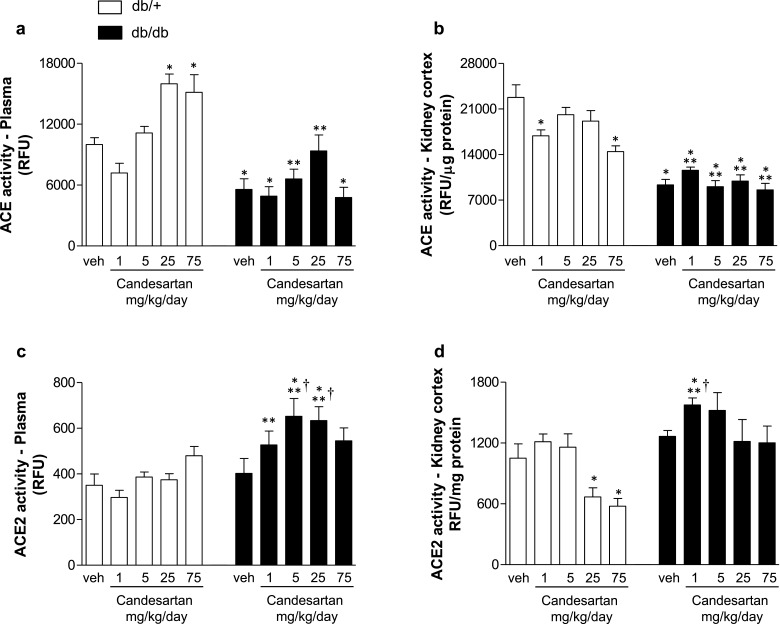
Intermediate dose of candesartan increases renal activity of ACE2, but not ACE, in db/db mice Enzymatic activity of ACE (**a**, **b**) and ACE2 (**c**, **d**) in plasma and kidney cortex from db/+ and db/db mice treated with vehicle or candesartan (1, 5, 25 or 75 mg/kg/d). Results are presented as mean ± S.E.M. of 5–7 mice. *P<0.05 compared with vehicle-treated db/+; **P<0.05 db/db compared with db/+ of corresponding candesartan dose; ^†^*P*<0.05 candesartan-treated db/db compared with vehicle-treated db/db.

Enzymatic activity of ACE and ACE2 was assessed in plasma and kidney cortex. Vehicle-treated db/db mice displayed lower ACE activity in plasma (Figure 7a) and kidney cortex (Figure 7b) compared with db/+ mice in basal conditions. Candesartan did not affect ACE activity in plasma or kidney from db/db mice. Plasma ACE activity was increased with higher doses of candesartan (Figure 7a) whereas kidney ACE2 activity was reduced with treatment (Figure 7b). ACE2 activity in plasma (Figure 7c) and kidney (Figure 7d) was similar in vehicle-treated db/+ and db/db mice. In db/db mice, candesartan at 5 and 25 mg/kg/d increased plasma ACE2 activity compared with vehicle-treated counterparts. In db/+ mice candesartan had no effect on plasma ACE2 activity, but reduced activity in kidney cortex at high and ultra-high doses.

### Effects of candesartan on renal MAPK phosphorylation

**Figure 8:**
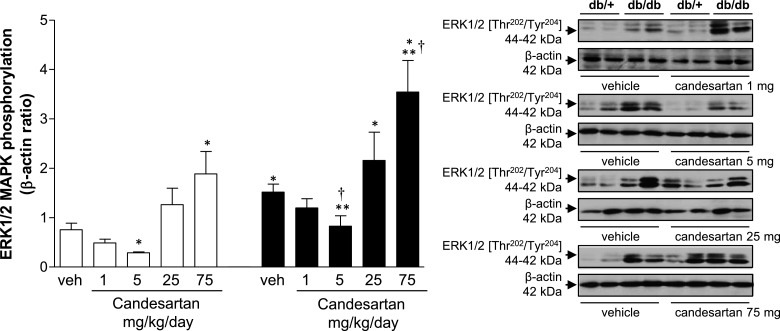
High and ultra-high doses of candesartan increase renal ERK1/2 phosphorylation in db/db mice Phosphorylation levels of ERK1/2 in kidney cortex from db/+ and db/db mice treated with vehicle or candesartan (1, 5, 25 or 75 mg/kg/d). Side panels, representative immunoblots of ERK1/2 [Thr^202^/Tyr^204^] and β-actin. Results are presented as mean ± S.E.M. of 5–7 mice. *P<0.05 compared with vehicle-treated db/+; **P<0.05 db/db compared with db/+ of corresponding candesartan dose; ^†^*P*<0.05 candesartan-treated db/db compared with vehicle-treated db/db.

Phosphorylation of renal ERK1/2 was increased in vehicle-treated db/db mice compared with controls (Figure 8). Candesartan at low doses, decreased ERK1/2 phosphorylation in db/db mice, whereas at ultra-high dose, opposite effects were observed. Similar responses were evident in db/+ mice. p38MAPK phosphorylation was increased in vehicle-treated db/db compared with db/+ mice (Supplementary Figure S5a). Candesartan, 25 mg/kg/d, amplified p38MAPK phosphorylation in db/db mice, whereas in db/+ mice, 75 mg/kg/d candesartan increased p38MAPK phosphorylation. In basal conditions, the magnitude of JNK phosphorylation was similar in db/+ and db/db mice (Supplementary Figure S5b). Treatment with increasing doses of candesartan was associated with an increase in JNK phosphorylation in db/db mice.

### Candesartan effects on renal pro-fibrotic and pro-inflammatory markers

**Figure 9:**
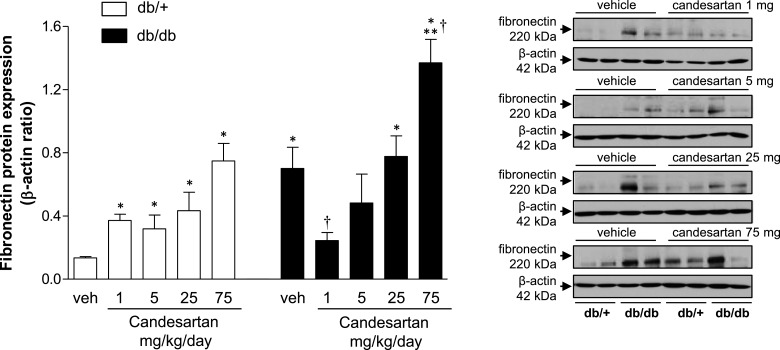
Candesartan modulates expression of pro-fibrotic and pro-inflammatory markers in kidney cortex from db/db mice Protein expression of fibronectin in kidney cortex from db/+ and db/db mice treated with vehicle or candesartan (1, 5, 25 or 75 mg/kg/d). Side panels, representative immunoblots of fibronectin and β-actin. Results are presented as mean ± S.E.M. of 5–7 mice. *P<0.05 compared with vehicle-treated db/+; **P<0.05 db/db compared with db/+ of corresponding candesartan dose; ^†^*P*<0.05 candesartan-treated db/db compared with vehicle-treated db/db.

Renal fibronectin expression was increased in vehicle-treated db/db compared with db/+ mice (Figure 9). Candesartan at 1 mg/kg/d reduced fibronectin content in db/db to similar levels observed in db/+ mice. However, at higher doses, candesartan increased fibronectin content in both strains. PAI-1 content was lower in db/db compared with db/+ mice in the vehicle-treated groups (Supplementary Figure S6a). Candesartan at 1 mg/kg/d increased PAI-1 expression in db/db mice. Kidney cortex from db/db mice displayed increased levels of VCAM-1 (Supplementary Figure S6b). Expression of the adhesion molecule was reduced by 75 mg/kg/d candesartan in both strains. No differences were detected in OPN expression between vehicle-treated groups (Supplementary Figure S6c). At 25 mg/kg/d candesartan increased OPN expression in db/db mice.

## DISCUSSION

Clinical studies demonstrated that in patients with persistent proteinuria, candesartan at doses four and eight times higher than the recommended daily dose of 16 mg for blood pressure lowering was associated with a significant additional reduction (>33%) in proteinuria [8,22,26]. Accordingly, doses greater than that originally recommended for hypertension have been suggested in the treatment of proteinuria [29–33]. Despite the encouraging findings, mechanisms underlying the added benefit of supra-high doses of candesartan remain unclear. Here we sought to address this by studying the putative role of the protective axis of the RAS, by focusing on ACE2, AT2R and Mas, in a mouse model of diabetic nephropathy treated with increasing doses of candesartan, from intermediate (1 mg/kg/d) to ultra-high doses (75 mg/kg/d). Major findings from our study demonstrate that in db/db diabetic mice, candesartan at intermediate to high doses (1) reduced albuminuria, (2) prevented renal injury evidenced by decreased mesangial expansion and glomerular hypertrophy, (3) reduced renal fibrosis and ERK1/2 phosphorylation, (4) increased renal expression of AT2R and Mas and (5) increased ACE2 expression/activity. Ultra-high doses of candesartan failed to protect against renal injury or albuminuria and did not influence ACE2, AT2R or Mas, but increased activation of MAPK and pro-fibrotic signalling in db/db mice. Our findings indicate that at intermediate-high doses, candesartan has renoprotective effects with associated up-regulation of the protective axis of the RAS, whereas at ultra-high doses, candesartan worsens renal damage and albuminuria without positively affecting on ACE2, AT2R and Mas in mice with diabetic nephropathy.

The RAS is an important therapeutic target for effective blood pressure lowering and for protection from progressive kidney damage in diabetic and nondiabetic kidney disease [2–14]. ARBs prevent or delay progression to end-stage renal disease independently of reductions in blood pressure [2, 3, 29–33]. This is particularly evident at high or ultra-high doses, where renoprotective effects of candesartan, and other ARBs, when administered at doses higher than those used in the treatment of hypertension, had added benefit without further affecting blood pressure [29–33]. ARBs at recommended blood pressure-lowering doses ameliorate kidney injury, glomerulosclerosis, kidney hypertrophy and prevent interstitial fibrosis, most likely by inhibiting Ang II signalling through the AT1R and by improving Ang II hemodynamic actions [6,8]. Mechanisms underlying the added blood pressure-independent benefit of high dose ARBs remains unclear but exposing the protective AT2R/Mas pathway, when AT1R is completely blocked, may be important. Previous studies demonstrated that ultra-high dose candesartan ameliorated renal injury and proteinuria in SHR through an anti-inflammatory mechanism by inhibiting NFκB signalling and chemokine production [33]. However, it is unclear whether this is due to AT1R inhibition or activation of AT2R and Mas. We suggest here that high dose candesartan exerts beneficial actions by up-regulating the counter-regulatory axis of Ang II–AT1R.

Albuminuria, an early and sensitive marker for progressive renal dysfunction, was a primary outcome in our study. Treatment with intermediate and high doses of candesartan reduced albuminuria indicating improved renal function. However at ultra-high doses, there was a progressive dose-dependent loss of the beneficial effect as evidenced in the db/db mice, which exhibited persistent albuminuria, and increased plasma creatinine and BUN/creatinine. The worsening of kidney function at ultra-high doses was associated with a significant decrease in blood pressure and it may be possible that the adverse renal effects at 75 mg/kg/d relate, in part, to significant blood pressure lowering and possible renal hypoperfusion. These effects might be related to excessive RAS blockade, as adverse events have been reported with combination therapy with ACE inhibitor and ARB in patients with diabetic nephropathy [28].

Diabetic db/db mice display many pathologic features at the functional and structural levels that are associated with the early phases of diabetic nephropathy, including albuminuria, mesangial expansion, reduction in glomerular cell number, as well as later changes, such as tubular damage and tubulointerstitial nephritis, effects that may be ameliorated by the candesartan treatment. Indeed, renoprotection with decrease in proteinuria have been reported in animal studies with higher doses of ARBs [32,33]. Candesartan at all doses was effective in normalizing mesangial expansion. Only the intermediate dose of candesartan showed recovery of tubular damage. These data suggest that intermediate dose of candesartan, which is still higher than the standard for blood pressure control, is beneficial for preventing the progression of diabetic nephropathy. However, ultra-high doses of candesartan induced more severe glomerular and tubular damage, effects that were exacerbated in db/+ non-diabetic mice. Lesions observed with ultra-high doses of candesartan are frequently encountered manifestations of drug-related injury, particularly in repeat-dose toxicity studies [37]. These severe structural changes were associated with functional impairment and probably relate to low blood pressure and possible hypoperfusion and ischemia.

To explore possible molecular mechanisms whereby candesartan induces renoprotective effects beyond AT1R blockade, we investigated the protective axis of the RAS, comprising ACE2/Ang-(1–7)/Mas receptor [15–19,38–40]. ACE2, a new member of the ACE family, predominantly metabolizes Ang II to generate Ang-(1–7). Ang-(1–7), in general, opposes actions of Ang II. By acting through the Mas receptor, Ang-(1–7) promotes vasodilatation and inhibits proliferation, inflammation and hypertrophy. Our study demonstrates that candesartan at intermediate to high doses increases ACE2 expression and activity in plasma and kidney. It is well-known that the levels of Ang II increase when the AT1R is blocked [41]. Thus, with ARBs, there is incomplete suppression of Ang II. It is possible that ACE2 amplification with intermediate to high doses of candesartan could be more effective by cleaving Ang II into Ang-(1–7), which further counteracts the actions of the AT1R activation. Thereby, ARB dose increment may provide organ protection beyond what is already achieved by the current blockade of AT1R. The effects of candesartan on ACE2 overexpression are associated with modulation of other RAS elements. The increase in AT2R and Mas receptor expression was combined with reduction in AT1R gene expression. Unexpectedly, kidney cortex from db/db mice displayed decreased ACE activity and expression. This could explain why db/db mice have normal levels of blood pressure, despite the dramatic vascular endothelial dysfunction extensively described in type 2 diabetes [42,43]. Nevertheless, candesartan did not affect ACE activity or expression. Overall these findings corroborate the notion that intermediate to high doses of candesartan may influence the protective components of the RAS to mediate the ARB blood pressure-lowering-independent renoprotective effects.

Activation of MAPKs is a pivotal signalling pathway involved in pro-fibrotic, pro-inflammatory and mitogenic effects in the kidney [12,13]. Previous reports demonstrated increased activation of MAPK in db/db [43,44], similar to our findings here. Candesartan variably attenuated activation of MAPKs, but at ultra-high doses, MAPK phosphorylation was amplified. This response is in line with the augmented deleterious renal functional and structural effects that we observed with ultra-high candesartan doses. One of the many processes influenced by MAPKs is inflammation, which is closely linked to renal fibrosis and complications of diabetic nephropathy [45–50]. The increased fibronectin protein content in kidney cortex from db/db mice was suppressed by candesartan at intermediate dose, indicating that at this dose candesartan has anti-fibrotic actions. Candesartan at 75 mg/kg/d mg was associated with increased expression of fibronectin, but not pro-inflammatory molecules VCAM-1, PAI-1 and OPN in db/db mice.

In summary, intermediate to high doses of candesartan ameliorate progression of nephropathy in db/db mice with associated up-regulation of the protective arm of the RAS. The present study provides new insights into some putative mechanisms, specifically activation of the ACE2/AT2R/Mas axis, whereby intermediate-high dose candesartan protect against diabetic nephropathy independently of blood pressure lowering. However at ultra-high doses candesartan loses its renoprotective properties and seems to promote more harm than benefit. This may relate to significant blood pressure lowering and possible renal hypoperfusion. Our findings may need to be considered in the clinical context where higher doses of ARBs are increasingly being used to treat diabetic nephropathy.

## Abbreviations

ACE2: angiotensin-converting enzyme 2
ACR: albumin:creatinine ratio
ARB: Ang II receptor blocker
AT1R: Ang II type 1 receptor
AT2R: Ang II type 2 receptor
BUN: blood urea nitrogen
OPN: osteopontin
PAI-1: plasminogen activator inhibitor-1
PAS: periodic acid–Schiff
RAS: renin–angiotensin system
SBP: systolic blood pressure
VCAM-1: vascular cell adhesion molecule-1
